# Aggressive Angiomyxoma—Report of a Rare Male Buttock Lesion

**DOI:** 10.1097/GOX.0000000000001879

**Published:** 2018-08-08

**Authors:** Frank Hsieh, Kai-Ti Chuang, You-Ting Wu, Chih-Hung Lin

**Affiliations:** From the * Department of Plastic & Reconstructive Surgery, Chiayi Chang Gung Memorial Hospital, Chang Gung University, Taiwan; † Department of Laboratory Medicine, Chiayi Chang Gung Memorial Hospital, Chang Gung University, Taiwan.

## Abstract

Aggressive angiomyxoma is a very rare benign tumor for male population with fewer than 50 cases reported since the description of this tumor. Most documented cases of aggressive angiomyxomas were found in genital, perineal, and pelvic regions in women of child bearing age. We report a case of a massive (> 20 cm) aggressive angiomyxomas in a man who presented with perineal swellings. Macroscopically the mass was highly vascular and lobulated with high similarity to plexiform neurofibroma. Microscopic examination revealed a hypocellular tumor comprising bland oval and spindle-shaped cells along with vessels of varying calibre. The accompanying stroma was myxocollagenous. Immunohistochemical staining showed CD34 and focal estrogen receptors positivity and negative staining for S100, actin, desmin, and progesterone receptors. The histologic and immunohistochemical features favored the diagnosis of aggressive angiomyxoma. Despite the rarity of such tumor in the male population, aggressive angiomyxoma should be considered in the differential diagnosis when encountering chronic para-perineal lesions.

Aggressive angiomyxoma is a rare, locally infiltrative mesenchymal tumor occurring mostly in women of productive age, but it is occasionally reported in men. It carries a high risk for local relapse, hence the need for differentiating it from the other mesenchymal perineal tumors. We report a case of a massive aggressive angiomyxoma extending from retro-pubic area anteriorly to buttock posteriorly.

## CASE REPORT

**Fig. 1.**
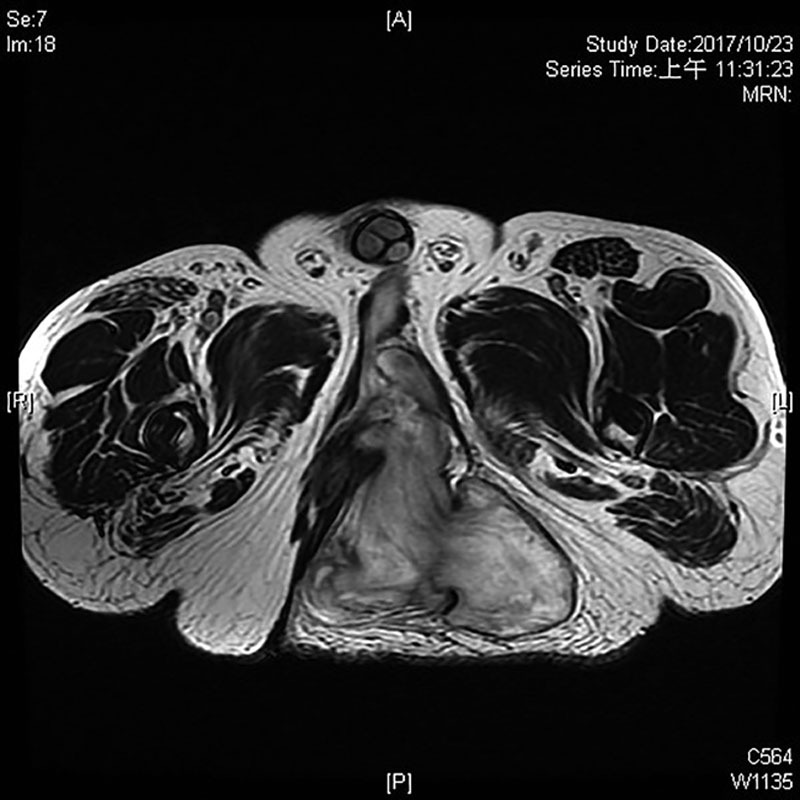
MRI axial view.

**Fig. 2.**
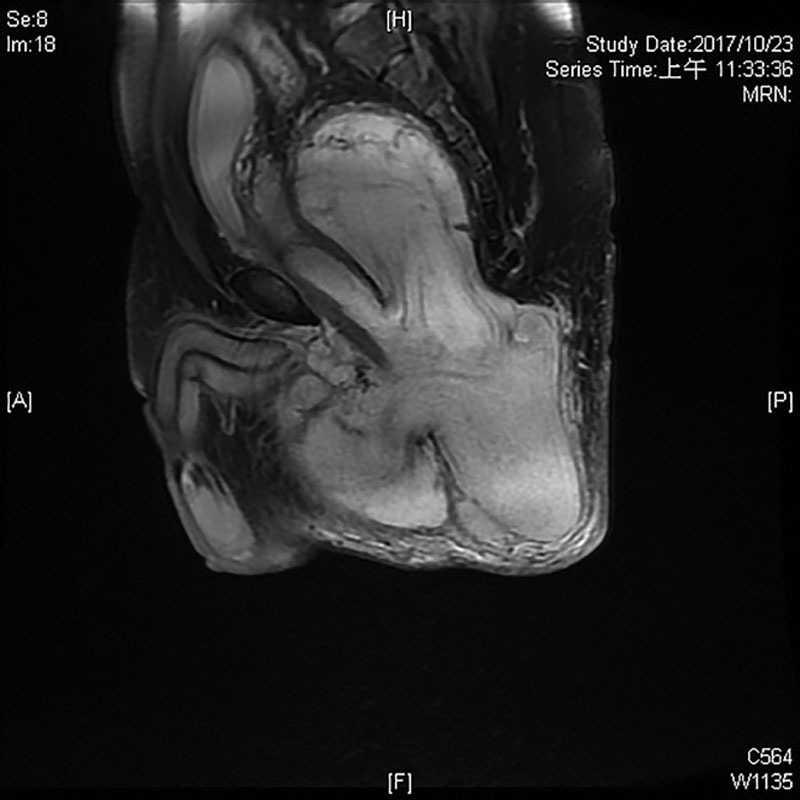
MRI sagittal view.

A 46-year-old man presented to our clinics with a mass located to his left buttock. He observed the gradual enlargement of this lesion for 2 years with no eliciting event. The lesion did not cause any neurological, urological, or colorectal symptoms. His main complaints were discomfort while sitting and embarrassment for cosmetic reason. The patient was a nonsmoker, nonalcohol drinker, and otherwise healthy with no family history of any cancer. Positive surgical history included a bilateral hip replacement 10 years ago post motor-vehicle accident complicated by multiple episodes of seroma. Physical examination showed a large left buttock mass with overlying intact skin and several neighboring scars from the previous hip surgery. It was soft, compressible, and nontender in consistency. Magnetic resonance imaging (MRI) from another hospital was reported as a large (20 × 10 × 10 cm) solid, lobulated lesion, spanning from retro-pubic anteriorly to left buttock posteriorly, well confined to subperitoneal space (Figs. 1, 2). As there was no local or regional lymph node enlargement on MRI, there was no suspicion of metastasis.

**Fig. 3.**
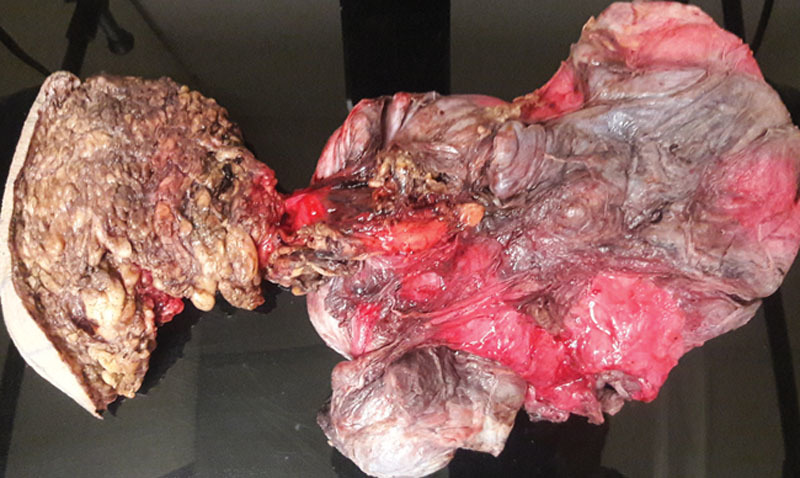
Grossly, the tumor is lobulated and poorly circumscribed with gray-tan and pink surface.

Patient underwent an en-bloc excision of the lesion with excess skin via gluteal incision. The lesion was lobulated and infiltrative. It had a distinctive appearance similar to plexiform neurofibroma (Fig. 3). The resection margin was the overlying capsule with all the tumor being completely excised. After ensuring the integrity of bladder and rectum in a multidisciplinary approach, the incision site was closed primarily with a drain.

**Fig. 4.**
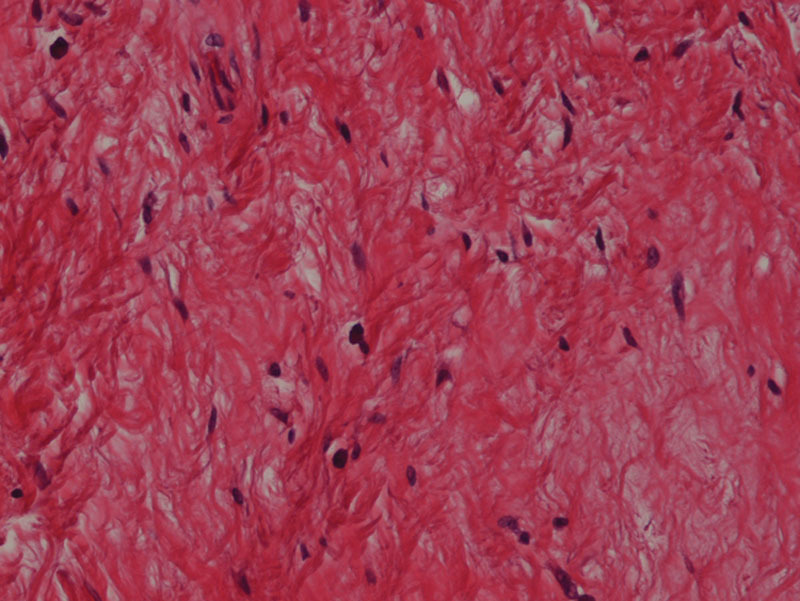
Microscopically, lesional cells are bland ovoid and spindled cells with negligible nuclear atypia (H&E stain, 200 ×).

Pathology reported as aggressive angiomyxoma. Macroscopically the specimen measured 23 × 17 × 10 cm. Microscopically, the tumor was composed of hypocellular, monotonous, and small spindled fibroblasts with no atypical mitotic figures (Fig. 4). Stroma was myxoid with collagen fibers and surrounded by dilated, thick walled vessels. Immunohistochemical stains revealed S100, desmin, and progesterone receptor negative with CD34 and focal estrogen receptor positive.

## DISCUSSION

Aggressive angiomyxoma is a rare mesenchymal tumor that most commonly arises in the vulvovaginal region, perineum, and pelvis of women. The tumor was first described by Steeper and Rosai1^1^ in 1983. Fewer than 350 cases have since been reported in the literature, with the female to male ratio 6:1.^2^ The sites that are involved in males in the limited case reports till date are scrotum, spermatic cord, inguinal region, prostate, and epididymis.^3^ The term aggressive emphasizes the often-infiltrative nature of the tumor and its frequent association with local recurrence after excision. It does not carry significant metastatic potential to suggest a malignant tumor. In the literature, 2 metastases (to lung and mediastinum) have been reported in women, but none in men so far.^4^ Patients often present with nonspecific symptoms that are frequently misdiagnosed with more common entities, such as a Bartholin cyst in females, lipoma, or hernia. Therefore, it is not uncommon for delayed presentation and most aggressive angiomyxoma is reported large (> 10 cm). In our case, the patient suspected the lesion was a complication of seroma after his hip replacement surgery and thus allowed its growth to 23 cm in diameter. Preoperative diagnosis is often difficult because of the rarity of these neoplasms and lack of specific features on imaging studies.^5^ Hence, most cases are diagnosed by histopathological examination after surgical excision.^5^

Histologic examination reveals a hypocellular and highly vascular tumor with a myxoid stroma containing cytologically bland stellate or spindled cells. Immunohistochemical stains demonstrate S100, desmin, and progesterone receptor negative with CD34 and focal estrogen receptor positive. Histologically, the hallmark of aggressive angiomyxoma is vessels of varying calibre haphazardly scattered throughout the parenchyma.^3,6^ Immunohistochemically, the majority of reported cases show positivity for desmin in the myoid bundles and/or stromal cells, whereas actins and CD34 may be variably positive.^2,7^ In female patients, the tumor cells are characteristically positive for estrogen and progesterone receptors, suggesting a hormonal role in the development of the tumor and some therapeutic advantage using hormone agonists has been proposed.^3^ The significance of association with female hormone receptor still remains unclear. The tumor did not stain consistently positive with these receptors reported in male patients,^4^ but might occur in some male series.^3^

The standard treatment for aggressive angiomyxoma is surgical excision, although achieving negative resection margins is difficult because of the infiltrative nature of the tumor and the often absence of a defined capsule.^6^ Interestingly, a review of 111 cases of aggressive angiomyxoma determining if radical resection is necessary by comparing patients’ risk of recurrence based on margin status. The data showed no statistical difference in remaining disease-free between groups of patients with positive and negative resection margin results (40% and 50% in 10 years, respectively).^8^ Even though complete surgical resection is the desired goal, incomplete removal is acceptable when significant operative morbidity is anticipated or when preservation of fertility is a concern.^8^ Hormonal therapy is recommended in the setting of neoadjuvant therapy after incomplete surgical resection.^7^ Recurrence mostly occurs within 5 years after complete surgical resection.^6^ Owing to the clear demarcation but proximity of rectum and anus, wide excision was not possible in this patient. No adjuvant or neoadjuvant therapy was offered in this case. However, considering the risk of recurrence careful follow-up and imaging modalities have been proposed.

## CONCLUSIONS

Aggressive angiomyxomas should be included in the differential diagnosis of large para-perineal tumors. This case signifies the extreme rarity of the tumor in males and highlights its typical histologic findings and its adequate management considering its locally infiltrative nature.
